# How is the artist role affected when artists are participating in projects in work life?

**DOI:** 10.3402/qhw.v11.30549

**Published:** 2016-05-09

**Authors:** Henrik Stenberg

**Affiliations:** Academy of Health and Welfare, University of Halmstad, Halmstad, Sweden

**Keywords:** Well-being, workplaces, visual artist, social interaction, emotional work

## Abstract

In Sweden, during the last decade, the artist has come to function as a creative resource in workplaces. There are two organisations, Skiss (Contemporary Artist in the Contemporary Society) and Airis (Artist in Residence), that organise projects for artists and coworkers. These projects are intended to have a positive effect on the well-being of organisations and their employees through artistic means, and the artist often focuses on the social interaction between the employees in their work. The artists’ work involves frequent interaction with coworkers. The aim of this article was to describe how visual artists’ roles as artists are affected by their engagement in artistic and social projects at workplaces in Sweden. The focus in the article is on the social interaction between artists and employees. The study is a qualitative narrative interview study with fine artists participating in different projects in work life. Since the artist's intervention is usually directed towards social relations in the workplaces, a social perspective on well-being is from a micro-sociological point of view. The categories in the interviews were how the artists worked with the projects, how the social interaction between artists and coworkers worked out, and how the artists evaluated the projects in relation to their ambitions. The results show that, many times, the artistic projects promote well-being in organisations and to some extent benefit the artist, but that the ability of the artists to actually function as artists can be problematic.

During the last decade in Sweden, as well as internationally, visual artists have come to function as a creative injection in organisations by undertaking artistic projects in collaboration with employees. The artist often works with relational art and focuses on the social environment at work. The purpose is to highlight and influence social relations and encourage employees to reflect on their work situation. When art and artistic methods are infused into the workplace, the hope is that people can participate in art on a daily basis and be positively influenced by art. The artist might contribute to an experience of meaningfulness that is not just short-lived, but that becomes established in the long term. Another purpose is to help artists to establish themselves in new fields in the labour market and further their development as artists and their artistic methods.

There are two main organisations that offer artists services in Sweden: Skiss (Contemporary Artist in the Contemporary Society) and Airis (Artist in Residence). More than 70 cultural workers have participated in projects in Airis since 2002 and more than 50 fine artists have participated in projects in Skiss since 2005. The organisations employ artists and educate them in areas such as cultural policies, health and welfare, project management, entrepreneurship and market analysis and also arrange the contact with the employer. The artists operate at the workplace for 8 to 10 months, half time or less, and usually work in a team with other artists (Airis, 2013; Skiss, 2013). Artists have been engaged in organisations such as schools, hospitals, elderly care, business and councils.

## Context: cultural policy and relational art

To be able to understand the artists’ working conditions in general, it is important to grasp how the present art field and cultural policy works. The use of the artist as a creative resource at workplaces is part of the current cultural policy in Sweden. This policy has changed over time. When the welfare state was at its peak in the 1960s and 1970s, the state policy was to support individual visual artists and artistic experiments with public funding (Duelund, 2008). Since public resources later became limited, the cultural policy promoted artists to be oriented towards the market or to be involved in projects with different collaborators in society, as in the described projects. Politicians want art to become more popular among citizens in order to engage people in artistic activities and events, a policy called “cultural democracy” (Duelund, 2008). Artists are thus supposed to be able to address a wider audience in their artwork. The artist sometimes becomes a social entrepreneur working in the civil sector with the ambition of influencing social relations (Stenström, 2008). This means that artists are oriented towards social life and involve other actors in their artistic work. The artists’ possible range of activity is changed as the artist is viewed as participating in and becoming a resource for society, rather than engaging in an internal artistic process that is basically a result of subjective artistic motives. Consistent with the current cultural policy, the ambition of Skiss and Airis is to widen the artistic field of work and promote collaboration between artists and other groups in society.

The change of the artist's role in society also depends on a changing view on artistry within the art field itself. Social influences have always been a prerequisite for artistry, but the visual artist was formerly regarded as a unique genius with an inherent ability to create, whereas the understanding of the social origin of artistic creativity was, to some extent, blurred (Pope, 2005). Today, the social impact on artistry is clearly recognized, and a sociological understanding of artistic creativity has moved forward (Bilton, 2010). Artistry is no longer considered separated from social life and from social relations; on the contrary, artistry is fully dependent on what takes place in social situations and in society (Sawyer, 2008). Artistry arises from relationships with others and through social participation. The art form called “relational aesthetics” implies a changed perception of art by which the artist has come to participate and be established in social life to a greater extent than before (Bishop, 2012; Lind, 1998; Stenberg, 2011). Relational aesthetics is a trend in the art field that has had a major influence on the visual arts from the mid-1990s onwards, and involves socialising, negotiating and connecting with others and inventing possible relations and alliances between separated individuals, groups and occurrences (Bourriaud, 1998). Relational aesthetics has motivated the artist to address the social environment by staging various projects, together with other actors, instead of being occupied with individual work in the studio and producing physical objects of different kinds in order to exhibit in art galleries. Relational art is, as is art in general, aesthetic, which means that the artist transforms the relational and social project into a material and visual form, and the art stands in dialogue with art history. The relational perspective in art means that the artist establishes a position in which he or she is in contact with the ongoing social life and at the same time preserves his or her integrity as an artist (Bourriaud, 1998).

## Health and well-being from a social perspective

It is well known that participation in cultural activities can promote psychosocial health. The production and acquisition of culture such as music, dance, theatre, literature and the fine arts has many beneficial impacts on individuals and groups. Findings show that participation in cultural activities, what is called “cultural welfare,” affects people positively (Sacco, 2013). For example, listening to music can be a means of reflecting upon and understanding personal moods and feelings (Skåland, 2013). In the case of visual art, it can help people to express and deal with different life experiences (Priebe & Sager, 2014) and, along with other measures, improve psychosocial health by strengthening the social capital among those concerned (Jensen, 2013). Those who experience art and culture can express positive outcomes emotionally, spiritually and socially, but it is not always evident how culture affects people (Georgieff, Lewis, & Rosenberg, 2009).

When it comes to improving health and well-being in organisations, each organisation can have different ambitions and different strategies to deal with these issues. Ambitions can be far-reaching or modest. The World Health Organization (WHO) definition of health is ambitious, stipulating that “[..] health is a state of complete physical, mental and social well-being and not merely the absence of disease or infirmity” (WHO, 2014). Sigurdson (2014) notes that the WHO definition, stating *complete* well-being, is utopian. Health is rather something that can be improved, using different tools and measures, to be good enough. A tool, among many, to strengthen health and well-being in organisations is to engage artists and other cultural workers in workplace projects. Since the artists concerned are often interested in understanding and coping with social relations at work, it is useful to utilise a social perspective on health. A basic understanding of well-being from a social perspective is the individual's ability to create and participate in close and emotional social bonds with other people (Scheff, 1990), and to be involved in meaningful social rituals with others (Collins, 2005). At the same time, the individual also needs to have access to a private mental space, what Winnicott (2005) calls a “potential space,” to be able to perform an “emotional work” that does not drain the individual's emotions in his or her interactions with other people (Hochschild, 2003, 2012).

## Research in the field

A number of researchers in disciplines such as sociology, health sciences, economics and occupational science have followed and evaluated several projects concerning artist involvement in workplaces. The research has primarily focused on how the artist-grounded projects have affected the workplace and the employees. In the case of Airis, research shows that the employees have often perceived the projects in a relatively positive manner, the artists have stimulated the workplace and interesting things have happened (Styhre & Eriksson, 2008). Eriksson (2008) found that managers and coworkers could leave their comfort zones during the projects and reflect on work and social relations in new ways. Styhre & Eriksson (2008) think that it is important that artists understand how they can affect working life in a positive manner and that organisations understand the artist's working terms. This is important since Lindqvist (2005) spotted that, in several projects, the artists’ skill as project leaders were more valued than his or her artistic knowledge.

Hellgren (2011) writes that artists within Skiss have been able to participate in changing work conditions, questioning the everyday practices that exist in each workplace. Augustinsson and Ericsson (2011) understand that most artists consider themselves appreciated at the workplaces, but not all as equal. Lindstrand (2010) finds that problems arising before the start of the projects have, in some cases, contributed to difficulties in working satisfactorily with staff. Karlsson (2011), who has followed Skiss projects for several years, states that the projects have positive effects on workplaces but that there is a tendency to focus on the salutogenic rather than the aesthetic outcomes. Widoff (2012), who has also followed the Skiss project, has observed that the project works best when artist and employees cooperate and frequently interact with each other. Widoff thinks this can be problematic since the employees are involved in the evaluation of the art. Artistic judgements normally arise within the art field itself.

Antal (2009) has studied evaluations of artistic projects in different countries. She finds that, in many reports, there is primarily a positive picture of the outcomes and that employees have learnt much about themselves and their colleagues during the projects. Antal (2009) stresses though that the research provides a vague picture of what actually comes out of the projects. Due to the anecdotal form of evidence, it is “[ ] difficult to distinguish between the effects that have really occurred and those which people would like to see occur” (Antal, 2009, p. 19). Some evaluations were directed towards the artists concerning what the projects meant for them. Also, these evaluations generally provide a positive image of what the artists have achieved and how they evolved artistically—apart from the practical problems of staging these kinds of projects, which do not always seem to have an apparent structure or a clear goal.

The evaluations usually focus on the outcomes of the projects and generalise what happens in them, but lack an extensive analysis of the relational dynamics and the interaction between artists and employees.

## Aim

The aim of this study was to describe visual artists’ experiences of how their role as artists is affected when they are engaged in artistic and social projects at workplaces in Sweden.

## Method

The methodological design is a qualitative study based on interviews with artists participating in projects in workplaces. The approach is hermeneutical phenomenology. Phenomenology in social sciences is characterised by an attempt to understand the individual life world and the meaning people sense in their actions and social relations (Bengtsson, 1998, 2001). The life world is the actual environment in which the individual participates that continuously transforms and can have different properties. Part of the artist's life world is the art field and the everyday interactions with other artists and actors in the field. Phenomenology is descriptive, and the intention of the study is to take part in the artist's narrative, postponing the evaluation of the respondent statements in order to be able to catch the multidimensional reality of which the artists are part.

Hermeneutics has similarities with phenomenology in trying to empathise with the individual and understand the meaning of his or her actions. In hermeneutics, the researcher has a pre-understanding of the field and interprets information on what is taking place. A hermeneutic approach means that the interpretation starts from the conceptions of the respondent and is posed against external factors (Warnke, 1995). The interpretation moves back and forth between the social elements and the totality, continuously increasing the understanding of the field (Ödman, 2007). The artist's narratives are interpreted in relation to the different contexts that affect their artistic ambitions, such as the projects concerned, the art field in terms of art history and contemporary art and the cultural policy. These contexts are also part of the artist's self-understanding.

### Participants

The study consists of interviews with six well-established contemporary visual artists, four women and two men of different ages. Two artists were connected to the project organisation Airis and four to the Skiss organisation. Contact was initially made with 10 artists and four of them declined to be interviewed for various reasons, mainly lack of time. The inclusion criterion was that the participants were well-established visual artists, since the study focus was on visual artists and their specific ways of working with relational art and other artistic methods, and not on other cultural workers such as actors, novelists, musicians and dancers that also participated in the Airis project. There was also a criterion that the artist had recently finished their project.

### Data collection

The research starting point was research in the field and the creation of the research question: How are visual artists role as artist affected when they are engaged in artistic and social projects at workplaces in Sweden? The research aim was operationalized into a questionnaire with open questions. The themes raised in the interviews have been the ways the artists worked during their time at the workplace, how they perceived that the staff embraced the activities that took place in the organisation, how they were influenced by the projects as artists, how the artists viewed the projects in an artistic perspective and whether they have developed artistically or not. Exploring these themes was important to be able to answer the research question. The interviews initially focused on descriptions of what happened in the projects, what is called “experience-near statements.” In the next step, the artist reflected upon their experiences in the projects from an organisational as well as artistic perspective, what is called “experience-distant statements” (Dalen, 2007). The interviews were carried out individually with each artist, apart from one interview containing two participating artists who worked together in a Skiss project. The interviews had a narrative structure and the artist was asked to talk freely about their work and their experience of the projects and their ability to work as an artist in this context (Johansson, 2005). In all interviews, the artists gave detailed information about specific projects, how the staff perceived them and their opinion of their role as artists in the project. The interviews lasted between 60 to 90 min. In some cases, I have taken account of the artist's images and texts that were part of their projects. The data also include seminars in Stockholm and Helsingborg, where various projects were presented and representatives from organisations concerned shared their experiences on the projects.

### Data analysis

The interviews were digitally recorded and transcribed verbatim by the interviewer. The analysis started with reading the data openly several times, understanding details and getting a sense of the wholeness in the respondent's narrative. The next step was to identify categories in the text in which correspondences as well as differences showed in the data material (Kvale, 2014). The main categories were how the artists worked with the projects, how the social interaction between artists and coworkers worked out and how the artists evaluated the projects in relation to their ambitions. The final step in the hermeneutic-inspired analysis was to relate the findings to the contexts that affect the artists and to micro-sociological theories suitable to understand the artist's well-being and working conditions. The corresponding themes in the analyses were how the cooperation between the artist and staff worked out, how the artist could maintain personal and artistic integrity in the projects and his or her evaluation of the projects from an artistic perspective.

### Ethics

The study has followed The Swedish Research Council ethical research principles. These are the informant requirement, the consent requirement, the confidentially requirement and the usage requirement. The participants in the study were verbally informed about the purpose of the study, and they all gave their consent to participate. They were informed that they would be anonymous and that they could abort the interview whenever they wanted. The artists are anonymous in the article and are assigned aliases. They were also informed that the information they gave was only to be used in scientific research and to become part of a scientific article.

## Findings

### The artist's work within the project and social interaction between artists and coworkers

Most artists engaged in workplaces have chosen to work with projects that involve the employees in collaboration and dialogues. Once the artists are situated at the workplace, they become acquainted with the staff and how employees perceive the work environment and are able to plan workshops and other events together with the employees. The ambition is often to strengthen the coworkers’ social bonds and community. Scheff (1990) considers social bonds a primary goal for humans. In modern society, individuals seek social bonds that are mutual and stable, but also leave room for individual needs. When a social bond is weakened, the individual can experience feelings of shame at not being accepted in the group. Some of the projects are explicitly initiated to improve the working environment. They consist of activities, games and events to inspire employees to relate to each other in new ways. Several of the artist workshops result in what Collins (2005) calls “interaction rituals.” Collins is using this concept to understand how meaning can be created when people come together in a mutual activity. Collins’ definition of an interaction ritual is that the participants know who is taking part in the ritual, and the participants are directed towards and concentrated on each other and on the task to be carried out. The ritual creates shared feelings and emotional energy that encourages the participants. At best, the shared feelings strengthen the individual's self-confidence and the solidarity within the group, not least because an increased value is invested in the symbols representing the group. The collective play, taking place in several of the projects, can be described as a type of creative and social gathering. Other activities are aimed at helping the employee gain new perspectives on his or her role at work and on identity as a whole. I will, further on, present some of the projects.

The artist Jane conducted a photography workshop in the City Planning Office in Gothenburg (CPOG), in which she and the staff together investigated the coworkers’ experience of their professional roles. Jane used a method in which existing images were combined with new images of the employees and were then manipulated in the computer program Adobe Photoshop. The employee could choose images from a picture library, which they thought described their professional role, and then changed the image in order to contribute to new perspectives on the role. One woman chose a picture of a boy who was hanging over a city from a rope. The woman then put on a mountain climbing harness that hung from a hook in the ceiling before shooting. The woman felt that she had an overview of the work, but she also felt inadequate to some degree. During this process, the employee could playfully view her identity and examine her professional role and self-image. The employee established a new perspective on herself in a straightforward way. If she got too close to uncomfortable emotions, she could say that she just did something funny.

Jane conducted yet another photo workshop at the Department for Construction Permits at CPOG, where the employees photographed each other when they rejected applications and when they gave consent. When they returned negative decisions, they were supposed to look sullen; when they gave consent, they were meant to look really happy in the photos. To look sullen, some of them bit on a lemon so that their faces wrinkled in a convincing manner. This seemingly simple workshop meant that the employees could find a playful distance to their work and to the responsible decisions they had to take. Playing with social roles and emotional expressions in this way is an opportunity to learn about their own role in the team and to strengthen the community and the social bonds at the workplace.

Personal feelings and reflections also occur in workshops in which the focus is explicitly on the individual. The artists Susan and Peter were operative in Region Skåne's Development Department (RUL, serving the southern part of Sweden) and staged a number of relational art-oriented projects at RUL. One project was staged at a 2-day conference arranged by RUL. The artists created a scenario in which the southern part of Sweden was flooded. The coworkers were instructed to build a dwelling place for the night or plan a place to stay overnight. A woman built a hut, which consisted of several rooms and a fireplace. During the workshop, the woman was overwhelmed with childhood memories of building huts. She explained that she was not accustomed to stopping and reflecting so much on her life; she usually steamed onward with work and daily activities. During the workshop, she remembered things from her childhood and felt, at the same time, that she was not living in the moment, which was a strong and overwhelming experience for her. Susan got a bit scared because the woman reacted so strongly, and Susan thought that she did not have the skills to take care of the feelings the woman expressed.

Julia was engaged in a supermarket in Gothenburg. She conducted a series of activities together with the employees in the store. One of the projects was to shoot short films based on the employees’ daydreams. The employees wrote down their daydreams that were then staged and filmed. One employee wanted to be in a nightclub. Another employee imagined that she was out riding. In the film, images of them carrying out their tasks at work were alternated with sequences from their staged daydreams. The films were, later, shown to the staff in the store.

Several of the artist workshops are meant to result in interactions between coworkers and to create a more intimate and personal community. Jane conducted a workshop at CPOG where 80 employees were divided into small groups, three in each group, and all groups were placed on mats on a big lawn. The staff would ask questions to each other to create a “My Friends Book,” as a child would. Questions included the following: What animal would you like to be? What is your favourite building? What do you want to be when you grow up? The answers were documented in a book that, later on, the employees could look at. The workshop was performed as an interactional ritual and created emotional energy among the employees.

Susan and Peter arranged a workshop at RUL called “Storytelling” at coffee breaks, during which the employees could tell personal stories. One coworker told a story about being shy as a child. In the school play, he had the role of a snowdrift. This satisfied him, since he was not particularly on show in the play, acting as a snowdrift. During the performance, his nose started to bleed and the snowdrift became red, and he suddenly became the focal point for the audience. Susan and Peter wrote a song based on this story called “Ballad of a Bleeding Snowdrift.”

Mark, who was placed at a unit in a hospital, noticed that the employees felt a low degree of meaningfulness at work and that the staff did not have the minimum level of commitment necessary to get them to accept the activities he suggested. Moreover, the staff had a stressful job and little time to participate in the projects. Mark, though, managed to get the staff to participate in some projects. In one of the workshops, he asked the employees to take photos of the interior of their homes, and later he put the photographs together into collages so that all could see the pictures. This gave rise to discussions about what the other colleagues’ homes looked like. By introducing activities, the coworkers were unaccustomed to, social relations were put into new situations and showed new perspectives.

Karen was placed at the town hall in Malmö and chose not to cooperate directly with the employees. Karen carried out six morning assemblies, conducted as performance. She introduced one of the performances by playing a piece of Finnish music and afterwards read parts from the Finnish author Zacharias Topelius’ book “The Story of our Country.” She wanted to give the employees an experience of Finnish history during coffee breaks that they could take with them and discuss during the day at work.

The artist used the dynamics in interaction rituals to be able to make unpredictable social processes occur in the workshops. By introducing activities that employees were not accustomed to, successful interactional rituals emerged and mutual creative thinking and action took place. When the employees were put in to new situations, new perspectives on work and social relations were established and social bonds were strengthened.

### The artists’ evaluation of the projects in relation to their professional ambitions

The artists gained different experiences concerning what they achieved in their projects. Several artists felt that the staff enthusiastically participated in the activities, which the artists initiated, and thought they were funny and creative elements at work. The artists believed that meaningful things happened in the workplace, in which employees perceived and reflected on their work situation and work relations in new ways. Susan and Peter felt that the staff enjoyed having the artists at the workplace, and they were offered an opportunity to continue their work after the project was finished. Similar to Lindstrand's (2010) findings, some artists initially had problems establishing the projects with employees, as Julia and Mark had. In Julia's case, the project was, in the end, appreciated by the employees at the supermarket. Jane's experience was that the project worked out well, and the staff at CPOG reflected on their working conditions. CPOG was transformed into a more customer-oriented workplace during this time. When the project was completed, criticism of work conditions was submitted to the management. Employees felt that, whereas the work environment used to be open and constructive, more recently the management style had become more authoritative. Jane's project helped the employees to discover that work could be more stimulating and creative.

Various answers were given about what the artists thought came out of their participation in the projects for their own sake as artists. Susan and Peter have generally positive experiences of the projects. Susan finds it more rewarding to work with others in different social situations than to produce art objects in the studio for an exhibition at a gallery. Susan and Peter are generally interested in the social processes that occur in these contexts. They have embraced the idea of relational aesthetics and do not see Skiss and Airis as institutional limitations of the artistic work but rather as a support for their artistic approach. They felt that they had freedom to do what they wanted in the projects, and they also received positive responses from employees. Julia thinks that, in many ways, some of her projects engaged the employees who participated in them, but that they initially found the projects to be quite demanding. Julia herself felt that she was primarily available for the sake of the personnel and identified an unspoken demand that her projects should have beneficial effects on the workplace. She constantly wondered about the purpose of her participation in the workplace, contrary to what she feels working with her own art. Mark initially had difficulty getting the staff involved in the projects and their commitment to the project was modest. Jane, in turn, had the opportunity to work in the City Planning Office with an artistic method that she has developed in other artistic contexts. As the project went along, she thought that her workshops worked well. However, the projects were so demanding in terms of guiding a great number of people (over 1 hundred), and organising activities, that her primary experience was leading the project rather than developing artistically. Jane believes that she has contributed to changes in the workplace with the help of her specific artistic skills—but at the same time, the interest in her as an artist has been minor. “They are not interested in the creation of art but in creative processes. This represents a romanticised image of the artist and not what the artist really is,” she says. Karen made similar remarks, saying that the management is not primarily interested in the art but in the artist's creativity and way of working. “This word ‘creativity’ has become some sort of buzzword. It is not the art they want but the creativity, the way we work, and it is supposed to be valuable for staff in the workplace. But who cares about art?”

To be engaged in outreach artistic projects of this kind involves emotional work in relation with those concerned. It can be stimulating and constructive emotional work, and the artist sometimes wants to work with emotional and social issues. But it can also be an emotional work that drains the artist of energy and creativity. Some of the artists felt a conflict in trying to work with artistic means and organising projects and supporting different needs among the employees. When this kind of conflict is prolonged, there is a risk of emotional dissonance. Hochschild (2003, 2012) uses the concept “emotional work” to describe how people in different situations in life have to manage their feelings, to express or suppress them, in relation to others. In private life, individuals can, at best, negotiate how to feel and how to express feelings that to some extent satisfy their emotional needs. In working life, especially working with people in the caring or service sector, the employee has to express certain feelings to satisfy the client, whether these feelings are consistent with one's own feelings or not. To cope with this kind of emotional work, the individual takes on a professional role in which the private and public roles are separated and the private self is used in a balanced way to meet clients’ demands. The consequences can be emotional dissonance and burnout, which naturally reduces well-being. Some artists find it hard to cope with the emotional work they had to perform during the projects.

## Discussion

The artists interviewed went through similar as well as different experiences in the projects they participated in, as is the case in other research. They were all able to use their artistic competence and to get employees to participate in the projects. Some of the artists felt appreciated by the employees for their personal commitment to the workplace, and they received a lot of confirmation and emotional response from them. The artists also found it valuable to work artistically in a workplace. Other artists met different obstacles trying to launch projects. The employees did not always fully embrace the project idea and were occasionally suspicious of the artist's intentions. Also, the employees’ working situation did not always permit full participation. Due to these and other problems, some artists found working with the employees quite demanding and they did not feel as if they were actually working as artists, rather as project managers or creative consultants, and the role as an artist turned out to be something other than what they expected and wanted it to be. The artists expressed that they prefer to work as artists in their own way rather than working in this kind of organisational context. These artists found that they were performing an emotional work in Hochschild's sense, trying to reconcile their own ideas and those of the employees about the projects’ content. When artists who are used to working with personal matters are involved in relational processes with employees and help to raise personal thoughts and feelings, they sometimes satisfy employees’ needs for emotional nourishment. To be able to keep up this approach, and not to face emotional burnout, it is important to have access to one's subjectivity, to highlight this in the artistic creation and protect the subjective drive against intrusion, sometimes by maintaining a certain distance from the surrounding social world. In other words, it is important to protect the intrinsic motivation against too much extrinsic motivation (Amabile, 1996; Amabile & Collins, 2010). Intrinsic motivation refers to personal interest, curiosity, satisfaction and positive challenge. Extrinsic motivation arises from demands outside the task itself and includes external directions and evaluation. The artist needs to establish what Winnicott (2005) describes as a “potential space,” permission to investigate the relation between intrinsic and extrinsic conditions, with limited judgements from others. The artist requires, just like people in general, a potential space to be able to be creative. The potential space can be constructed in seclusion as well as in interaction with others, depending on the artist's need. When artists are working with staff on various events in the workplace, at best they carry with them a certainty about who they are and what they want as artists and can thus offer the workplace new perspectives on what is happening there. The artist's intention is to make the private and personal more present in the organisation, which can be related to their specific professional role as artists. Artists often view their professional role as contradictory to instrumental roles, and to what Hochschild calls “emotional rules,” instructions for using emotions in labour, by highlighting different aspects of being human. As the artist in his or her work relies to varying degrees on their own subjectivity, he or she creates a dialogue between a personal and a public role. Artistry in itself can be understood as a relation between individual and social processes that differs between each artist (Hennessey, 2003). The role of the artist should not be romanticised, but the artist's working conditions can be different from conditions in working life in general. Gauntlett writes that the artist's work is, to different degrees, rooted in her or his own personal inclinations that has some kind of “[..] autobiographical and expressive dimension” (Gauntlett, 2007, pp. 30–33). In some cases, this means that their private experiences are portrayed in art, which of course need not always be the case (Stenberg, 2002). Visual artists occasionally want to work with art subjectively such that authenticity, emotions, personal reflections and free expression are important, as they were during the modern epoch (Lee, 2006). The visual artists usually want to be creative in a way they choose themselves, that differs from organised artistic activities, what Bilton (2010) calls “manageable creatvity” and social engagements, and therefore it is hard to formulate a general model, a fixed break-even point, where artistry best occurs. A series of social processes affects the ability to be and feel creative. Cultural policy performed on different levels in society is part of manageable creativity, and it wants to influence the artist and the art field with its interests, and this can cause tensions between the artist and the cultural policy.

Many contemporary artists want to work with artistic projects in direct relation with other actors. The relational aesthetics mean that the artist, to a greater extent, placed the exploration of the relationship between the individual and social environment in a direct interaction with diverse social situations and actors. The projects arranged by Skiss and Airis can be described as an institutionalised form of relational aesthetics. The purpose of Skiss and Airis is that the artists are active in an organisation on its own premises. This means that the role of the artist, as it is perceived in contemporary art, includes that the artist announces something particular that is art, and needs an artistic freedom in his or her work to bring a new perspective on social life and what it means to be human (Bishop, 2012). To be in contact with artistic creativity, and simultaneously be emotionally and practically engaged in outreach projects, demands a certain distance towards others, the possibility to stand outside of the workplace, otherwise the artistic integrity is at risk. Some of the interviewed artists managed to preserve their artistic integrity, while others did not manage to do that to a satisfactory degree.

## Conclusion

Artist participation in projects in work life is a consequence of the prevailing cultural policy, which aims to involve artists in society and to give them new opportunities to earn their living and also encourage people in general to get involved in cultural activities. Art and culture should support a citizen's integration into society and work life, as is the ambition of the artistic projects. Artistry is perceived as a resource for society and not merely as an internal artistic affair. Since art has long defined its position as being relatively autonomous compared with other areas in society, there is a clash between these perspectives on art that also can be seen in the interviews with the artists. Participation in the projects does not always lie up to their artistic aspirations. Contemporary art is involved in society from the specific position of being able to reflect on and criticize social conditions. The contemporary artist explores habitual beliefs, breaks social taboos and uncovers society's sore points. Bishop (2004, 2012) observes that artists are commissioned to repair deficiencies in society instead of the changes that take place on a structural level, such as improving the welfare systems or changing employees working conditions. When artistry is brought down to the level of a mainstream conception of art, there is a risk that artistry becomes diluted as a force to produce new perspectives and meanings (Oakly, 2009). Bishop is critical towards relational art when the primary aim is to stage social gatherings. Art's mission, she thinks, is to provoke thinking and reflection, and the artist has an active role in this to a significant extent, as what can be called a heroic creative. “Heroic creative” means that the artist, to some extent, is the carrier of a subjective heroic creative narrative that can influence others to be creative (Bilton, 2010). Some artists manage to be that heroic creative, and to keep their integrity as artists in collaboration with employees, but this is not always easy due to different relational circumstances.

Karlsson (2011) considers Skiss not an artistic project, but an intervention through artistic praxis. In the projects, there is a possible extension of the aesthetic spheres. Karlsson identifies that the increasing demands in working life affect the employees’ health and well-being, and artist involvement in the workplace can be part of the pursuit of a better working environment. At the same time, he thinks there must be room for artistic particularity and a possibility for artists to not always cooperate with others.

In terms of what has emerged from this study, it can sometimes be problematic to operate as an artist in workplaces and simultaneously perpetuate artistic integrity. It can be asked whether artists should be performing this kind of work. If so, in order to improve the positive outcomes of the projects, it is important to prepare coworkers and the organisation for collaboration with the artist, which has not always been the case, and to ensure that the artist can actually work in an artistic way that is appropriate for him or her.
